# Phylogenetic evidence supporting the nonenveloped nature of hepadnavirus ancestors

**DOI:** 10.1073/pnas.2415631121

**Published:** 2024-10-29

**Authors:** Jaime Buigues, Adrià Viñals, Raquel Martínez-Recio, Juan S. Monrós, José M. Cuevas, Rafael Sanjuán

**Affiliations:** ^a^Institute for Integrative Systems Biology, Universitat de València and Consejo Superior de Investigaciones Científicas, València 46980, Spain; ^b^Institut Cavanilles de Biodiversitat i Biologia Evolutiva, Universitat de València, València 46980, Spain; ^c^Department of Genetics, Universitat de València, València 46100, Spain

**Keywords:** pararetroviruses, virus evolution, metagenomics

## Abstract

Reverse-transcribing animal DNA viruses include the hepadnaviruses, a well-characterized family of small enveloped viruses that infect vertebrates but also a sister group of nonenveloped viruses more recently discovered in fish and termed the nackednaviruses. Here, we describe the complete sequence of a virus found in the feces of an insectivorous bat, which encodes a core protein and a reverse transcriptase but no envelope protein. A database search identified a viral sequence from a permafrost sample as its closest relative. The two viruses form a cluster that occupies a basal phylogenetic position relative to hepadnaviruses and nackednaviruses, with an estimated divergence time of 500 My. These findings may lead to the definition of a “proto-nackednavirus” family and support the hypothesis that the ancestors of hepadnaviruses were nonenveloped.

Two major families of nonintegrated reverse-transcribing DNA viruses have been defined by the International Committee for the Taxonomy of Viruses: *Caulimoviridae* (1) and *Hepadnaviridae* (2). While caulimoviruses infect plants, hepadnaviruses infect vertebrates, including humans. In particular, human hepatitis B virus (HBV) is a leading cause of cirrhosis and hepatocellular carcinoma worldwide (3). In 2017, a new group of viruses distantly related to hepadnaviruses was identified in teleost fishes (4), and related viral sequences have since been reported (5). These viruses may represent a viral family called the nackednaviruses, or *Nudnaviridae* (2). Their genome is slightly smaller than that of hepadnaviruses (2.7 to 3.1 kb versus 3.0 to 3.4 kb), and importantly, they do not encode an envelope protein. The characterization of nackednaviruses suggested an ancient origin for hepadnaviruses from nonenveloped ancestors in fish (4).

In a recent study, we described DNA virus sequences from fecal samples of different bat species sampled in Spain (6). Analysis of the raw data from this study led us to identify a viral contig with an ambiguous taxonomic classification within the order *Blubervirales*. This sequence was obtained from the feces of a single *Myotis scalerai* individual captured on June 2022 in the Sima de la Higuera, a cave near the village of Pliego in the province of Murcia, Spain. PCR and sequencing of the cytochrome B gene confirmed that this sample was from *M. scalerai*, an insectivorous species (7). The viral contig corresponded to a complete circular genome of 3,472 nt, as it had terminal redundancy, and was sequenced with an average coverage of 150 reads per base. Its identity was then confirmed by PCR using sequence-specific primers that allowed us to amplify the entire viral genome in overlapping fragments.

This virus, which we called Pliego virus, contained two open reading frames (ORFs) of 1,149 and 2,376 nt homologous to, but highly divergent from, the capsid/core (C) and polymerase (P) of hepadnaviruses and nackednaviruses. The predicted C protein of Pliego virus was considerably larger than that of hepadnaviruses and nackednaviruses (382 aa versus 180 to 260 aa). Protein BLAST (BLASTp) analysis indicated that the closest sequences for the C and P proteins corresponded to HBV (Genbank accession ANQ89943.1) and fish-associated HBV (WAQ80622.1), respectively. However, the presence of multiple stop codons in the S-congruent reading frame revealed a typical nackednavirus feature. The Pliego virus genome contained three additional putative ORFs unrelated to those of other nackednaviruses or hepadnaviruses (Fig. 1*A*), but the structure and function of the corresponding proteins could not be predicted.

**Fig. 1.**
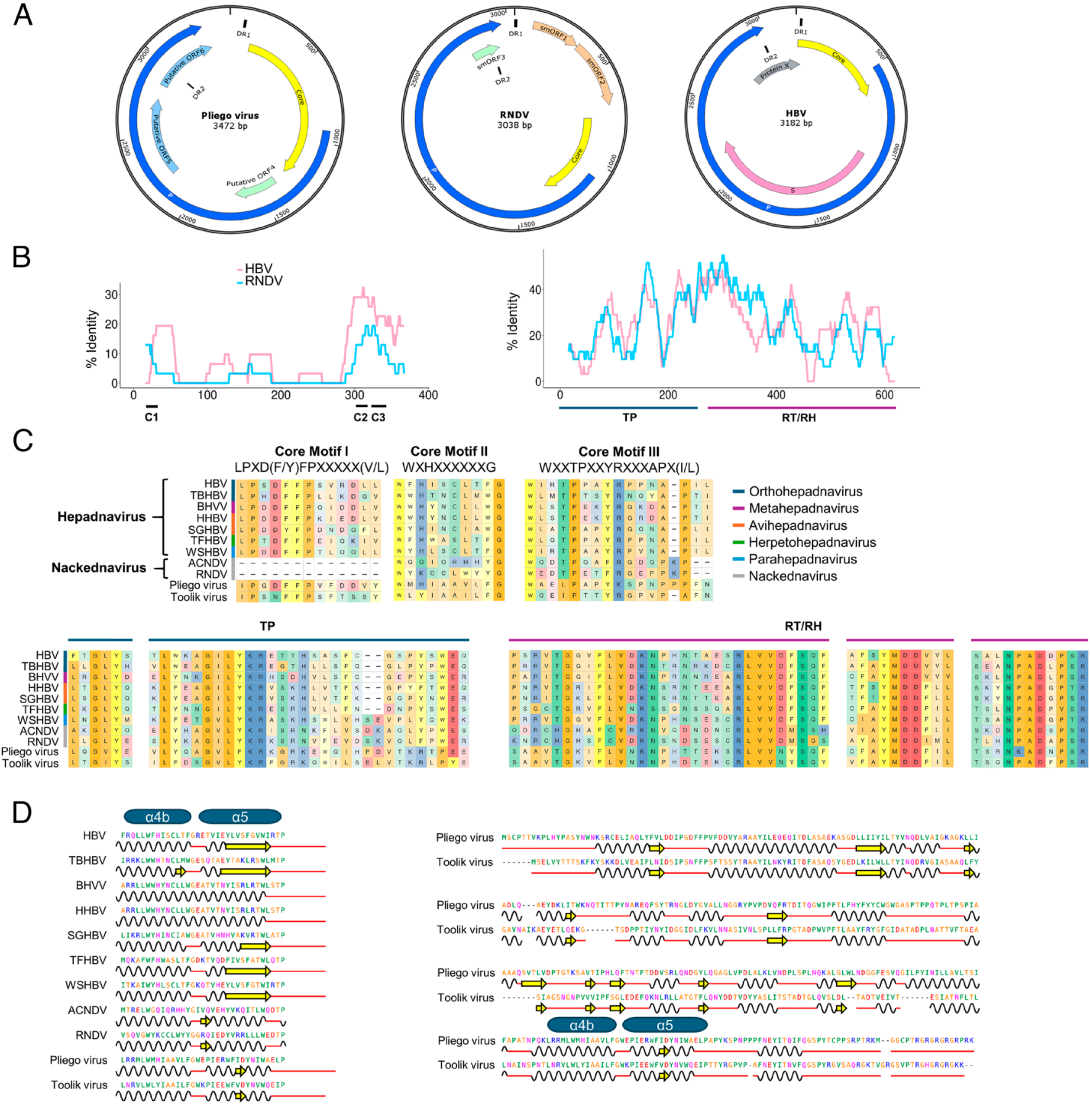
Comparative genome annotation. (*A*) Genome organization of Pliego virus, RNDV, and HBV. In addition to genes with known function, putative ORFs, and two short direct repeats of sequence AACTTCTACTGCAC (DR1 and DR2) are indicated. (*B*) Similarity between the amino acid sequences of Pliego virus and RNDV or HBV for the C (*Left*) and P (*Right*) proteins, using a 30-residue sliding window. Core conserved motifs CI-CIII, terminal protein domain (TP), and reverse transcriptase and RNaseH domains (RT/RH) are shown. (*C*) Alignments of the three core conserved motifs (mapping to residues 36 to 48, 303 to 312, and 326 to 341 of the C protein in the Pliego virus sequence) and selected regions of P domains for seven representatives of different hepadnavirus genera, two nackednaviruses, Pliego virus, and Toolik virus. Notice that, in the GLY conserved motif of TP, L is substituted for V in Pliego virus and I in Toolik virus. In contrast, the tyrosine-methionine-aspartate-aspartate (YMDD) motif of the RT/RH is fully conserved. (*D*, *Left*) Alignment of the C protein α-helices α4b and α5 for representatives of hepadnaviruses and nackednaviruses, along with Pliego and Toolik viruses. (*D*, *Right*) conservation of the predicted secondary structure along the protein C of Pliego and Toolik viruses. Black waves, yellow arrows, and red lines correspond to α-helices, β-sheets, and loops, respectively.

A similarity plot of ORF C against a representative nackednavirus (rockfish nackednavirus, RNDV) and HBV showed a very high divergence (10.5% and 11.9% overall amino acid identity, respectively, Fig. 1*B*). Nevertheless, we were able to identify conserved core motifs of vertebrate HBVs (8), which were more similar to those of hepadnaviruses than to those of nackednaviruses (Fig. 1*C*). Phyre 2 and mapping of predicted secondary structures to the sequence alignment also suggested conservation of the core α-helix 4b found in hepadnaviruses and nackednaviruses (4, 9) and, to a lower extent, of α-helix 5 (Fig. 1*D*). The P protein was less extremely divergent than C (23.8% and 18.3% overall amino acid identity with RDNV and HBV, respectively; Fig. 1*B*). In the *Blubervirales*, P contains a reverse transcriptase (RT; IPR00477) and RNaseH (IPR001462) domain, as well as a terminal protein (TP; IPR000201) domain involved in the initiation of reverse transcription (10), all of which were detected (Fig. 1*C*). However, the RNA element epsilon, whose secondary structure is essential for the priming of reverse transcription (11), was not found. Similar to nackednaviruses (4), the spacer between the TP and RT found in hepadnaviruses was shorter than that in hepadnaviruses. Other genomic motifs conserved in nackednaviruses and hepadnaviruses were identified, such as the direct repeats (DR1 and DR2) containing the TATA box and the polyadenylation signal.

We set out to detect similar sequences in databases. To this end, we first searched two large bat metagenomics projects (PRJNA953205, PRJNA929070), but this did not yield any hit. We then carried out a BLASTp analysis of the C and P proteins in IMG/VR4, an extended database of uncultivated virus genomes (12). This revealed an unpublished 3,316 nt viral sequence (BK068391) assembled in a metagenomics study of permafrost soil carried out in the Toolik Field Station, Alaska. This “Toolik virus” shared amino acid sequence identities of 34.5% and 43.4% with Pliego virus C and P proteins, respectively, and also lacked a gene encoding an S protein. Despite the high divergence between the Pliego and Toolik sequences, the predicted secondary structure of the C protein showed a remarkable conservation (Fig. 1 *C* and *D*).

We then inferred the phylogenetic relationships between Pliego virus, Toolik virus, hepadnaviruses, nackednaviruses, and other reverse-transcribing viruses (Fig. 2*A*). In this analysis, we also included HEART insect endogenous retroelements related to *Blubervirales* (13). The RT phylogeny showed that nackednaviruses and hepadnaviruses formed well-supported sister clades, whereas the Pliego and Toolik viruses formed a separate cluster that occupied a basal position relative to the nackedna/hepadnavirus group. Following previous work (4), we also obtained a time-calibrated Bayesian tree of the P protein sequence (excluding HEART elements), using an endogenous avihepadnaviral element (eAHBV-FRY) integrated into the Neoaves genome for calibration (4, 14, 15). We estimated a divergence time of about 450 My between nackednaviruses and hepadnaviruses, similar to the value obtained in previous work (4), while Pliego and Toolik viruses would have diverged more than 500 Mya (Fig. 2*B*). We propose that the order *Blubervirales* should include hepadnaviruses, nackednaviruses, and the Pliego/Toolik clade, which we tentatively designate as “proto-nackednaviruses.” Moreover, our results support the hypothesis that ancestral nonintegrated reverse-transcribing animal DNA viruses were nonenveloped and that the S ORF in hepadnaviruses probably arose by genetic overprinting (4).

**Fig. 2.**
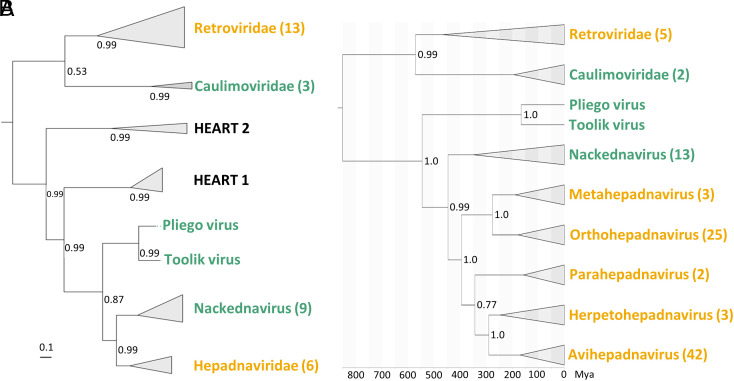
Phylogenetic position of the newly described cluster. (*A*) Bayesian phylogenetic tree of the RT domain. The Pliego and Toolik sequences were added to the alignment used in a previous work (13) including blubervirus-related insect HEART retroelements. Scale bar, number of amino acid substitutions per site. (*B*) Time-calibrated Bayesian tree of the P protein. The Pliego virus and Toolik virus sequences were added to the alignment used in a previous work (4). Scale bar, Mya. Nodes are collapsed by genus or family, and the number of sequences in each clade is given in parentheses. Enveloped viruses are shown in yellow and nonenveloped viruses in green. Numbers at branching nodes indicate posterior probability values.

According to the above tree, proto-nackednaviruses might have diverged from other *Blubervirales* around the Cambrian. Hence, the rapid radiation of early metazoans during this time might have been accompanied by diversification of bluberviruses. However, uncertainties in estimated divergence times, cross-species transmission events, and the fact that many viruses remain undiscovered complicate evolutionary inferences. Cross-species transmission is generally thought to be rare among DNA viruses (16, 17) but has been suggested for bat hepadnaviruses (18) and fish nackednaviruses (19).

In contrast to nackednaviruses, which have been found in teleost fish (4, 5), the identified proto-nackednaviruses are unlikely to be fish viruses since they were obtained from the feces of an insectivorous bat and permafrost soil. We speculate that these might be arthropod viruses, but alternative possibilities cannot be ruled out at present. Further research is warranted to test whether Pliego or related viruses could infect bats, as bats are a known source of primate hepadnaviruses (20).

## Methods

Bats were captured and identified at the species level, and fresh fecal samples were collected in accordance with European and Spanish regulations. Extracted nucleic acids were used for cytochrome B Sanger sequencing and de novo sequencing on a NextSeq instrument. Contigs were assembled and used for viral sequence detection. PCR amplification and Sanger sequencing were performed to confirm the Pliego virus sequence. Sequence annotation included ORF search and prediction of protein domains, structure, and function. Sequence diversity and phylogenetic analyses were performed using R, Biostrings, and Bayesian inference.

## Supplementary Material

Appendix 01 (PDF)

## Data Availability

Previously published data were used for this work (6). The viral contig described in this study was deposited in Genbank under accession number PQ119727 (21). Other data are available at NCBI BioProject (22, 23).
