# Supplementary material for: Phylogenetic evidence supporting the nonenveloped nature of hepadnavirus ancestors
Source: Proc Natl Acad Sci U S A. 2024 Oct 29;121(45):e2415631121. doi: 10.1073/pnas.2415631121 (PMC11551314; doi:10.1073/pnas.2415631121)
Supplement: Supplementary file 1 — Appendix 01 (PDF) [file pnas.2415631121.sapp.pdf]

# Supporting Information for

# Phylogenetic evidence supporting the nonenveloped nature of hepadnavirus ancestors

Jaime Buigues[1], Adrià Viñals[2], Raquel Martínez-Recio[1], Juan S. Monrós[2], José M. Cuevas[1,3]*, Rafael Sanjuán[1,3]*

[1]Institute for Integrative Systems Biology (I2SysBio), Universitat de València and Consejo Superior de Investigaciones Científicas, València, Spain

[2]Institut Cavanilles de Biodiversitat i Biologia Evolutiva, Universitat de València, València, Spain

[3]Department of Genetics, Universitat de València, València, Spain.

*Corresponding authors: cuevast@uv.es, rafael.sanjuan@uv.es

**This PDF file includes:**

Supporting Text

SI References

## Supporting Text

### Extended methods

**Bat capturing.** Samples were obtained in a recent study as described previously (1). Briefly, individual bats were captured using nylon mist nets and a harp trap, identified visually to the species level, sexed, measured, weighed and briefly placed in cotton bags to recover fresh fecal samples. Bats were captured in accordance with the European directive regulating the protection of animals used for scientific research (2010/62/EU, Article 1), subsequently transposed into Spanish legislation (Royal decree 53/213, 1 February, Article 2). The procedures followed in this study (i.e. capture, non-invasive handling and in situ release of wild animals) are not under the status of animal experimentation and hence do not require an IACUC approval document, but instead specifically a permit from the competent regional authority (Ref. Exp. 2022-VS (FAU22_009)).

**Sample processing.** Samples were collected in tubes containing phosphate-buffered saline, kept cold initially, and then at -20 °C until they were transported to the laboratory and stored at -80 °C for further processing. Fecal samples were homogenized using ceramic beads and supernatants were filtered through a 1.2 µm pore size. Filtrates were used for total nucleic acid extraction and extracts were stored at -80 °C. For host taxonomic confirmation, we amplified by PCR and Sanger sequenced a 148 bp region of the cytochrome B gene using specific primers as described previously (2).

**Pliego virus sequencing.** Sequencing libraries were prepared using the Nextera XT DNA kit and subjected to paired-end sequencing in a NextSeq 550 device with a 150 bp read length at each end. Reads were deduplicated, quality filtered with a quality trimming threshold of 20, and reads below 70 nucleotides in length were removed. De novo sequence assembly was performed using SPAdes v3.15.4 (3) with the meta option, and MEGAHIT v1.2.9 (4) using default parameters. Contig taxonomically classification was performed with Kaiju v1.9.0 (5). Virsorter2 v2.2.4 (6) was used to detect viral sequences, which were confirmed using CheckV v1.0.1 (7). Coverage statistics were calculated by remapping the trimmed and filtered reads from the sequencing library using Bowtie2 v2.2.5 (8). To confirm the sequence and completeness of Pliego virus genome, the following specific primers were designed to PCR-amplify six overlapping fragments of approximately 700 bp: 1F (5´-CGCGCTTATTCATGCTCA), 1R (5´-TTTGTGGTGGTGTTGGTGAT), 2F (5´-TCACACAAGGGTGGATACCAT), 2R (5´-TTCTTCGGGAGTTCCATACG), 3F (5´-CAAGAAAAATGGGAGGATGC) and 3R (5´-GACCGTCGGATATGGTGAGT), 4F (5´-TATCATCAGCCGCAGTAACG), 4R (5´-ACCCGATAAGTCGTTGATGG), 5F (5´-ACGAGACAAGGCAACCAAAT), 5R (5´-TTCGGTTGTGTCCATTTTCA), 6F (5´-CCCGTGCATTTCGTCTCTAT), and 6R (5´-CAGGCAATCAAGCGTTACAA).

**Sequence analysis and phylogenetic reconstruction.** ORFs were predicted using ORFfinder (ncbi.nlm.gov/orffinder) and protein domains were annotated using InterProScan v5.63-95.0 (9) with the Pfam database v35.0. The C proteins and ORFs with missing protein domains were also analyzed with Phyre2 (10) to predict protein structure conservation. Similarity plots for C and P protein alignments (sliding window of 31 residues) were created using R 4.4.0 and Biostrings v2.70.2 (https://bioconductor.org/packages/Biostrings). Mapping of predicted secondary structures to protein sequence alignments were obtained with Ali2D, implemented in the MPI Bioinformatics Toolkit (11). A Bayesian tree of the RT was performed using MrBayes 3.2.7a (12) using a LG+G substitution model. A total of two million generations with four chains were run sampling every 1000 generations the posterior trees. The consensus tree was inferred burning the first 25% of posterior trees. To obtain a time-calibrated tree of the P protein, the Pliego virus and Toolik virus P sequences were aligned to those used in a previous study (13). The less conserved regions in the global alignment were removed using TrimAL v1.2rev59 (14) with the gappyout option, resulting in a final alignment of 490 positions. JTT+G4 was selected using ProtTest v3.4.2 (15) as the best-fitting amino acid substitution model. A time-calibrated Bayesian tree was computed with BEAST v2.7.6 (16), using a calibrated Yules speciation prior and an optimized relaxed clock model with log-normal distribution. As previously described (13), a uniform mean clock rate and a normally distributed prior with mean of 69.2 and standard deviation of 1.735 for the age of the eAHBV-FRY root were used. The final time-calibrated tree was computed combining three independent analyses using a chain length of 50 million each.

**SI References**


1. J. Buigues, *et al.*, Full-genome sequencing of dozens of new DNA viruses found in Spanish bat feces. *Microbiol. Spectr.* e0067524 (2024).
2. A. Lopez-Oceja, D. Gamarra, S. Borragan, S. Jiménez-Moreno, M. M. de Pancorbo, New cyt b gene universal primer set for forensic analysis. *Forensic Sci. Int. Genet.* **23**, 159–165 (2016).
3. S. Nurk, D. Meleshko, A. Korobeynikov, P. A. Pevzner, metaSPAdes: a new versatile metagenomic assembler. *Genome Res.* **27**, 824–834 (2017).
4. D. Li, C.-M. Liu, R. Luo, K. Sadakane, T.-W. Lam, MEGAHIT: an ultra-fast single-node solution for large and complex metagenomics assembly via succinct de Bruijn graph. *Bioinforma. Oxf. Engl.* **31**, 1674–1676 (2015).
5. P. Menzel, K. L. Ng, A. Krogh, Fast and sensitive taxonomic classification for metagenomics with Kaiju. *Nat. Commun.* **7**, 11257 (2016).
6. J. Guo, *et al.*, VirSorter2: a multi-classifier, expert-guided approach to detect diverse DNA and RNA viruses. *Microbiome* **9**, 37 (2021).
7. S. Nayfach, *et al.*, CheckV assesses the quality and completeness of metagenome-assembled viral genomes. *Nat. Biotechnol.* **39**, 578–585 (2021).
8. B. Langmead, S. L. Salzberg, Fast gapped-read alignment with Bowtie 2. *Nat Methods* **9**, 357–359 (2012).
9. P. Jones, *et al.*, InterProScan 5: genome-scale protein function classification. *Bioinforma. Oxf. Engl.* **30**, 1236–1240 (2014).
10. L. A. Kelley, S. Mezulis, C. M. Yates, M. N. Wass, M. J. E. Sternberg, The Phyre2 web portal for protein modeling, prediction and analysis. *Nat. Protoc.* **10**, 845–858 (2015).
11. F. Gabler, *et al.*, Protein sequence analysis using the MPI bioinformatics toolkit. *Curr. Protoc. Bioinforma.* **72**, e108 (2020).
12. F. Ronquist, *et al.*, MrBayes 3.2: efficient Bayesian phylogenetic inference and model choice across a large model space. *Syst. Biol.* **61**, 539–542 (2012).
13. C. Lauber, *et al.*, Deciphering the origin and evolution of hepatitis B viruses by means of a family of non-enveloped fish viruses. *Cell Host Microbe* **22**, 387-399.e6 (2017).
14. S. Capella-Gutiérrez, J. M. Silla-Martínez, T. Gabaldón, trimAl: a tool for automated alignment trimming in large-scale phylogenetic analyses. *Bioinforma. Oxf. Engl.* **25**, 1972–1973 (2009).
15. D. Darriba, G. L. Taboada, R. Doallo, D. Posada, ProtTest 3: fast selection of best-fit models of protein evolution. *Bioinforma. Oxf. Engl.* **27**, 1164–1165 (2011).
16. R. Bouckaert, *et al.*, BEAST 2.5: An advanced software platform for Bayesian evolutionary analysis. *PLoS Comput. Biol.* **15**, e1006650 (2019).
